# Acoustic Behavior of Hollow Blocks and Bricks Made of Concrete Doped with Waste-Tire Rubber

**DOI:** 10.3390/ma9120962

**Published:** 2016-11-26

**Authors:** Esteban Fraile-Garcia, Javier Ferreiro-Cabello, Beatriz Defez, Guillermo Peris-Fajanes

**Affiliations:** 1 Engineering Data Mining And Numerical Simulations (EDMANS) Group, Department of Mechanical Engineering, University of La Rioja, 26004 Logroño, La Rioja, Spain; javier.ferreiro@unirioja.es; 2 Centro de Investigación en Tecnologías Gráficas, Universitat Politècnica de València, 46022 València, Spain; bdefez@upv.es (B.D.); gperis@upv.es (G.P.-F.)

**Keywords:** acoustic behaviour, building elements, waste-tire rubber, doped concrete, used tires, circular economy

## Abstract

In this paper, we investigate the acoustic behaviour of building elements made of concrete doped with waste-tire rubber. Three different mixtures were created, with 0%, 10%, and 20% rubber in their composition. Bricks, lattice joists, and hollow blocks were manufactured with each mixture, and three different cells were built and tested against aerial and impact noise. The values of the global acoustic isolation and the reduction of the sound pressure level of impacts were measured. Results proved that highly doped elements are an excellent option to isolate low frequency sounds, whereas intermediate and standard elements constitute a most interesting option to block middle and high frequency sounds. In both cases, the considerable amount of waste-tire rubber recycled could justify the employment of the doped materials for the sake of the environment.

## 1. Introduction

Traditionally, waste has been managed towards its burial in landfills, with or without previous incineration and/or compression. Nevertheless, this is no longer a satisfactory practice, since it is well-known that natural resources are limited and pollution levels are threatening the current way of life. An interesting new approach to minimizing waste and reducing the consumption of resources consists of reincorporating industrial waste and by-products to the manufacturing processes of the same or other products. This is the base for achieving a so-called “circular economy” [1,2,3].

Examples of circular economy with regard to building materials are increasingly popular. For instance, ethylene-vinyl acetate (EVA) generated in the manufacturing of footwear is used to improve the thermal behaviour of non-structural concrete [4], and sawdust timber together with low-density poly-propylene is employed to produce masonry bricks with enhanced acoustic absorption rates [5].

Following the same philosophy, using waste-tire rubber as an aggregate in the manufacturing of building elements could both contribute to the preservation of the environment and meet the official requirements set for building elements [6,7,8]. In some specific cases, they could even improve the performance of traditional elements.

Commonly, building elements such as bricks, lattice joists, and hollow blocks are made of ceramics, plaster, or concrete. In the case of ceramic and plaster elements, previous studies have analysed the possibility of incorporating waste-tire rubber with success [9,10,11,12]. In the case of concrete elements, research has been focused on incorporating different materials (including end-of-life tires) to investigate selected features [13,14,15,16]. Rubberized concrete has shown a superior strain modulus compared to regular ceramic bricks, and therefore an improved behaviour towards seismic actions [17,18]. It has also been verified to be more noise-absorbent than regular concrete. Therefore, it is an excellent candidate for the construction of highway noise barriers [19,20] and asphalt pavement [21].

Further works have analysed of the durability and safety of the concrete–rubber mixtures. The analyses have proven the suitability of the concrete–rubber mixture as structural and non-structural material [22,23,24,25,26]. A suitable model is required to describe the behaviour of concrete doped with rubber in engineering applications. Evolutionary polynomial regression (ERP)—a new evolutionary technique of data mining—has been used to forecast the mechanical properties of concrete doped with rubber [27].

In this research, we investigate the acoustic behaviour of waste-tire rubber concrete bricks (wall elements), lattice joists, and hollow blocks (unidirectional slab elements) with different proportions of rubber in the mixture. Geometrically identical cells were built with the elements made of every mixture. Therefore, we assess the acoustic performance of bricks, lattice joists, and hollow blocks jointly, and not as separate components, since they work together in real buildings. The acoustic performance of building elements is influenced by several complex factors. Hence, it is necessary to validate any constructive new proposal with specific tests, such as the one previously described [28].

## 2. Materials and Methods

### 2.1. Materials

Raw materials were initially stored in different silos. Later, they were mixed homogeneously by means of an industrial kneader for 3 min. Three different mixtures were created. Namely, M1, M2, and M3. Each mixture had a different percentage of waste-tire rubber, and therefore a different density, as displayed by Table 1. As rubber is less dense than conventional concrete, the density of the mixture decreases as the rubber content increases.

**Figure 1:**
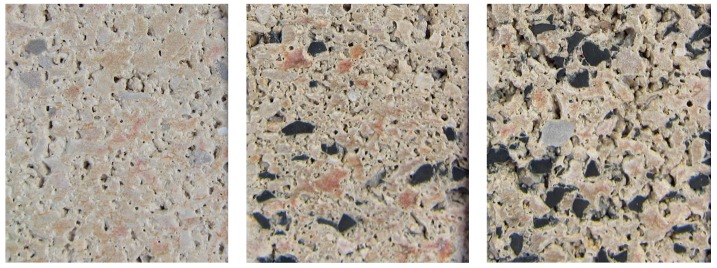
Mixtures: M1 (**left**); M2 (**centre**); M3 (**right**).

These waste-tire rubber percentages were established as the most convenient by a previous study [6]. The doped particles of rubber are visible in Figure 1.

**Figure 2:**
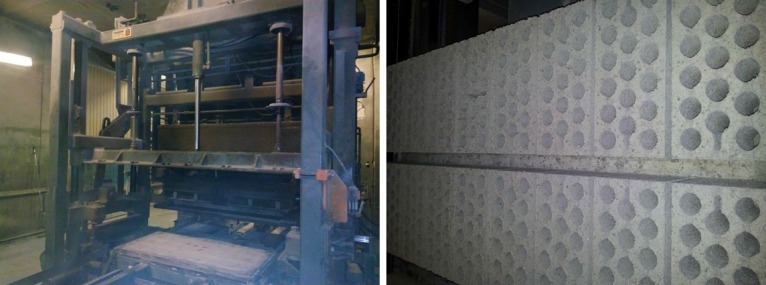
Brick manufacturing: compacting unit (**left**); and tray conveyor (**right**).

**Figure 3:**
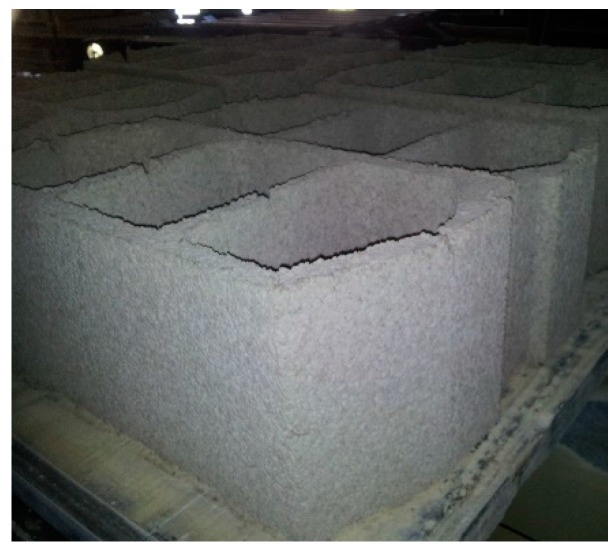
Hollow block manufacturing.

To manufacture the bricks and hollow blocks, the fresh mixture was poured into the corresponding mold and pressed by an automatic compacting unit (model PB-1200, Balbinot, Bellegarde-Sur-Valserine, France) at 69 KPa for 5 s. The mixture was vibrated simultaneously until the elements were removed from the mold. Then, the elements were brought to the curing chamber by means of a tray conveyor (see Figure 2 and Figure 3).

**Figure 4:**
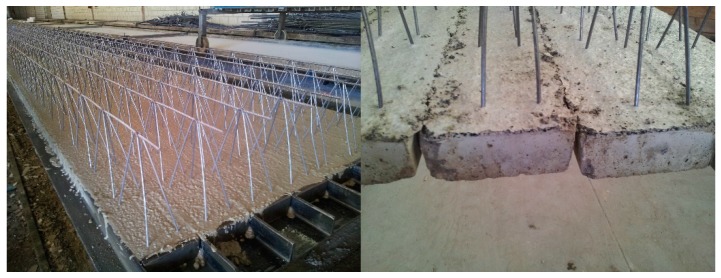
Lattice joist manufacturing: recently incorporated rubber layer (**left**); and after consolidating (**right**).

Lattice joists were originally made of conventional concrete (code HA-25-B-12-IIa). Scrap-tire rubber was incorporated as a top layer to obtain an elastic support for the hollow blocks (see Figure 4).

### 2.2. Cell Geometry and Composition

Commonly, the acoustic properties of a new material are assessed by testing individual samples according to the standards (ISO, ASTM, etc.). This kind of assessment is required, and reveals essential information about the performance of the material. However, the assessment of the material in real performance conditions should be considered in order to acquire a deep knowledge of the tangible potential of the material. Therefore, three cells (rooms) were built with the elements manufactured with every mixture—namely, cells C1, C2, and C3. This is an interesting new approach to the acoustic evaluation of new materials.

**Figure 5:**
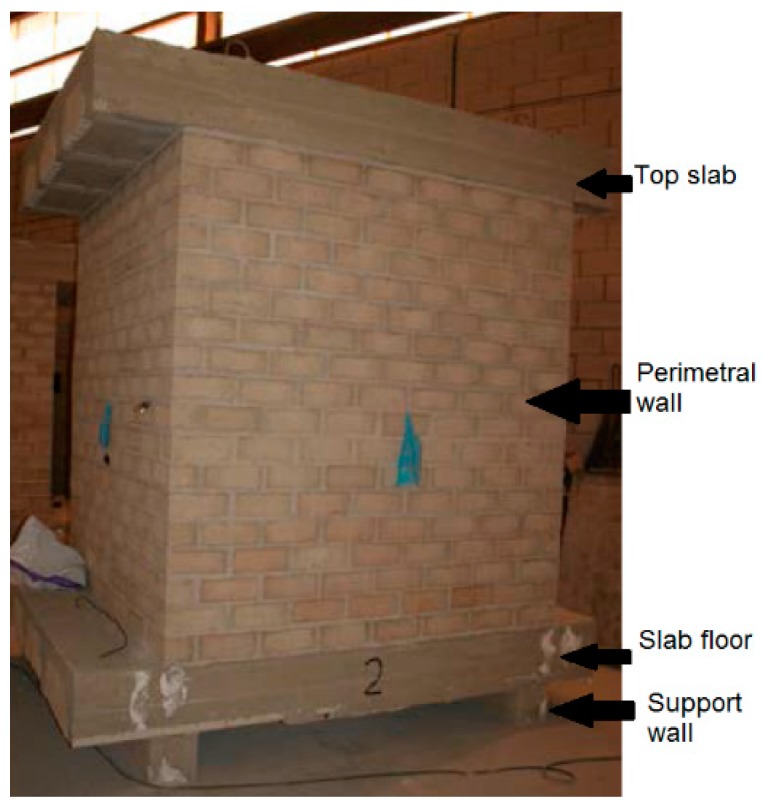
Geometry of the cells.

The three cells had equal external dimensions (2.00 m × 2.00 m × 2.60 m). Each one was made of two short support walls, a unidirectional slab floor, four perimeter walls at 90°, and a top slab. As shown in Figure 5, the support walls sat over the shop floor, the slab floor rested over the support walls, the perimeter walls were raised over the slab floor, and finally, the top slab was laid on top of the perimeter walls. An entrance in the shape of a rectangle was initially opened in one of the walls to allow for the introduction of the measuring equipment. The entrance was then closed for measurement with the same building elements. Results were collected by a computer outside of the closed cells.

The support walls were made of 20 cm-thick concrete blocks. The perimeter walls were made of 10 cm-thick prefabricated bricks. Both slabs were 30 cm thick. They were made of the prefabricated lattice joists and hollow blocks. Conventional (non-doped) concrete mortar was used to build every component of the cells. Table 2 summarizes the correlation between the cells and the mixtures.

Cells were built inside a large industrial warehouse (dimensions significantly higher than the dimensions of the cells).

### 2.3. Equipment

The following equipment was used in order to carry out the acoustical measurements:

- Sound analyser BRÜEL & KJAER, model 2260.
- Sound calibrator BRÜEL & KJAER Type 4231.
- Noise generator BEHRINGER ULTRACURVE, model DSP-8000.
- Tapping machine BRÜEL & KJAER, model 3207.
- Standard microphone.
- Measurement equipment were all properly certified.

### 2.4. Methodology

Airborne and impact sound insulation were considered to investigate the acoustic behaviour of the three cells. The pair of parameters *D*_nT_ (Standardized Level Difference) and *D*_nT,w_ (Weighted Standardized Level Difference); and Ln (Normalized Impact Sound Pressure Level) and *L*_n,w_ (Weighted Normalized Impact Sound Pressure Level) were measured to assess each type of sound isolation. As previously commented, these are “in-situ” measurements, ideally adapted to the geometry of the cells and the characteristics of the environment.

Measurements were taken in the frequency band interval from 50 to 5000 Hz. Nevertheless, only the results of the interval between 100 and 3015 Hz were assessed. Study frequencies were 100, 125, 160, 200, 250, 315, 400, 500, 630, 800, 1000, 1025, 1006, 2000, 2005, and 3015 (one-third octave bands). Thus the spectral adaptation coefficients could be considered. Comparisons were made in one-third octave bands, so that they could be conducted in more detail.

A minimum of six readings were considered to calculate the average of the different parameters. One position of the loudspeaker and three positions of the microphone with two readings each were carried out.

ISO standards 140-3 [29], 140-4 [30], 140-5 [31], 140-7 [32], 140-9 [33], 140-10 [34], 717-1 [35], and 717-2 [36] were taken as references for this acoustic evaluation. Nevertheless, the particular characteristics of the building elements and cells made the literal application of such standards impossible, and several modifications of the standard’s procedures were necessary. Therefore, conclusions were based on the comparison between the responses of the three different building elements of this particular research.

All measurements were taken in the daytime. Background noise was measured and compensated to avoid the interference of external sources. Temperature and moisture were measured to assure that their values were compatible with the specifications of the equipment.

Readings were taken by applying sampling methods of the sound pressure with intervals of 10 s, selected within the evaluation time span.

At the beginning and end of each reading, the sensitivity of the equipment was verified with the sound calibrator, according to standard IEC 60942:2003 [37]. A deviation no higher than 0.3 dB from the initial reference value was guaranteed.

#### 2.4.1. Reverberation Time

For the measurement of the reverberation time, standard ISO-3382 [38] was used, employing the method of the integrated impulsive response because of its simplicity. The range of fall in use was always larger than 20 dB, but small enough to tend to a straight line. At the end of the range, the range of fall must be larger than 10 dB over the background noise. The pulse source must be able to generate a level of peak acoustic pressure so that the falling curve started at least 45 dB above the background noise of the corresponding frequency band.

**Figure 6:**
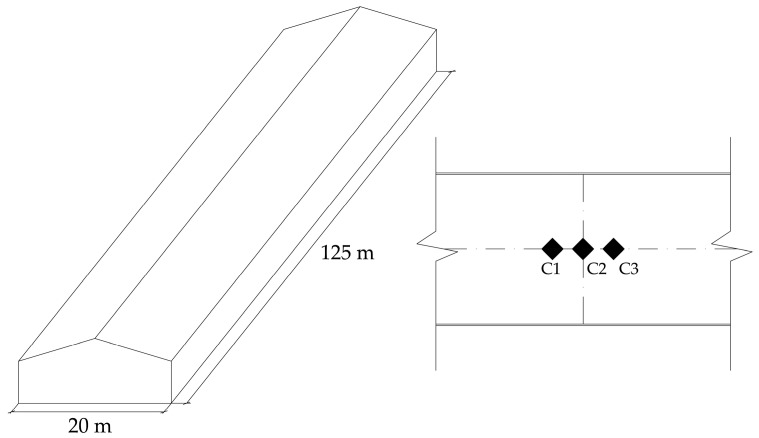
Geometry of the warehouse and location of cells inside.

The volume of the industrial warehouse (13,500 m^3^) was taken for the calculation of the absorbent surface. In the case of the airborne sound insulation, reverberation times were so large (due to the dimensions of the warehouse) that they were not considered. Figure 6 represents the geometry of the warehouse and the location of the three cells inside.

#### 2.4.2. Airborne Sound Insulation

The assessment of the airborne sound isolation was done by means of parameters *D*_nT_ and *D*_nT,w_. The method of the difference between the emitted level and the transmitted level employing dB(A) was used, since the possible absorption of the warehouse (receiver enclosure) had to be considered as a constitutive part of the isolation of the cell (transmitter enclosure).

The sound source was located inside the cell, so that a fuzzy acoustic field was created, with level differences no higher than 6 dB between third octave adjacent bands. Two speakers were used, located at two opposite corners of the experiment wall. Both speakers sat on elastic bases, to avoid solid transmission of sound. The sound created was stationary and had a continuous spectrum in the range on the considered frequencies, which provided a proper signal–noise correlation in the high frequencies of the warehouse. The power of the sound created was high enough so that the sound pressure in the warehouse was at least 10 dB higher than the background noise in any frequency band.

**Figure 7:**
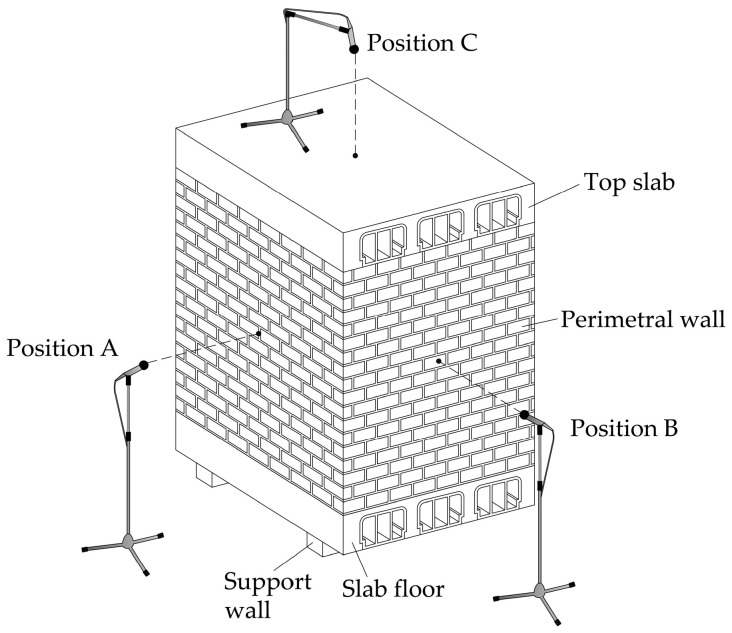
Location of the microphone with respect to the cells.

A microphone was located in the warehouse to capture the transmitted sound. Three different positions of this microphone were considered for readings (Figure 7):

- Position A: at 1.50 m from the centre of perimeter wall A (wall parallel to the main direction of the lattice joists of the slabs).
- Position B: at 1.50 m from the centre of perimeter wall B (wall perpendicular to the main direction of the lattice joists of the slabs).
- Position C: at 1.50 m from the centre of the top slab.

#### 2.4.3. Impact Sound Insulation

The assessment of the impact sound insulation within interior enclosures was done by means of parameters *L*_n_ and *L*_n,w_.

**Figure 8:**
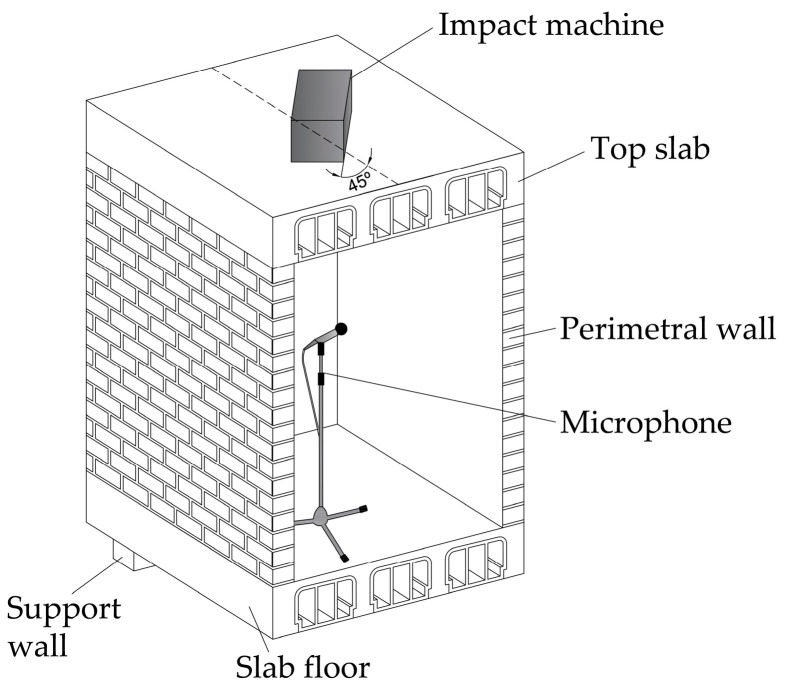
Measurement of the impact sound insulation.

The impact machine was located over the middle point of the top slab, at 45° to the main direction of the lattice joists. The transmitted impact sound was read using a microphone located in the geometrical centre of the inner volume of the cell (Figure 8).

The relative humidity and temperature of the warehouse were registered. They met the technical requirements indicated by the equipment manufacturer. Readings were taken only after the sound created by the impacts was stationary.

## 3. Results

The main objective of the research was to assess the acoustic behaviour of waste-tire rubber concrete as building material. The procedure described here does not follow any particular standard, but three cells were built and tested. Therefore, relative results are more relevant than absolute results. In other words, the results of cell C1 (0% rubber) were taken as the benchmark, and the performance of cells C2 and C3 were compared to them. According to this approach, two ratios were calculated:

Relative improvement of the Standardized Level Difference:
  RID_nTC*i*_ = (*D*_nTC*i*_ − *D*_nTC1_)/*D*_nTC1_
and Relative improvement of the Normalized Impact Sound Pressure Level:
  RIL_nTC*i*_ = (*L*_nTC*i*_ − *L*_nC1_)/*L*_nC1_

On the other hand, it is worth remarking that we observed that in all study cases and measurements, background and source noise were grouped into a band of ±1.5 dB. Therefore, the readings were very meaningful.

### 3.1. Airborne Sound Insulation

**Figure 9:**
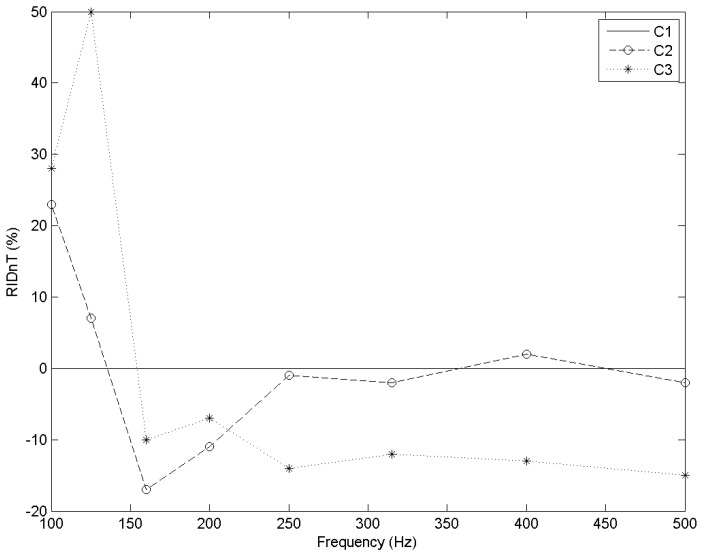
RID_nT_ (%) vs. frequency (Hz) for each cell type. Frequency range 100–500 Hz.

With regard to airborne sound insulation, cell C3 showed the best improvement for sound frequencies between 100 and 140 Hz approximately (Figure 9). Therefore, it is convenient to isolate very low frequency sounds, such as low frequency instruments (bass guitar, bass drum, etc.) or low frequency road traffic vehicles (heavy trucks, tractors, etc.).

Cell C1 presents the best results for frequencies between 150 and 500 Hz (Figure 9). Those are the frequencies of fundamental male and female voices.

**Figure 10:**
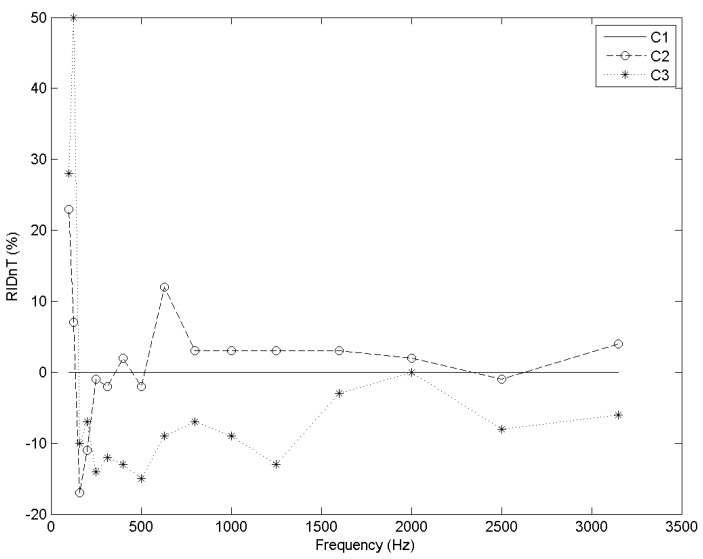
RID_nT_ (%) vs. frequency (Hz) for each cell type.

Cell C2 is the best solution for frequencies higher than 500 up to 2500 Hz. Particularly, cell C2 has a remarkable performance at 630 Hz (Figure 10).

For frequencies higher than 2000 Hz, differences between C1 and C2 are too narrow to differentiate between them, whereas cell C3 presents a poor response (Figure 10).

### 3.2. Impact Sound Insulation

**Figure 11:**
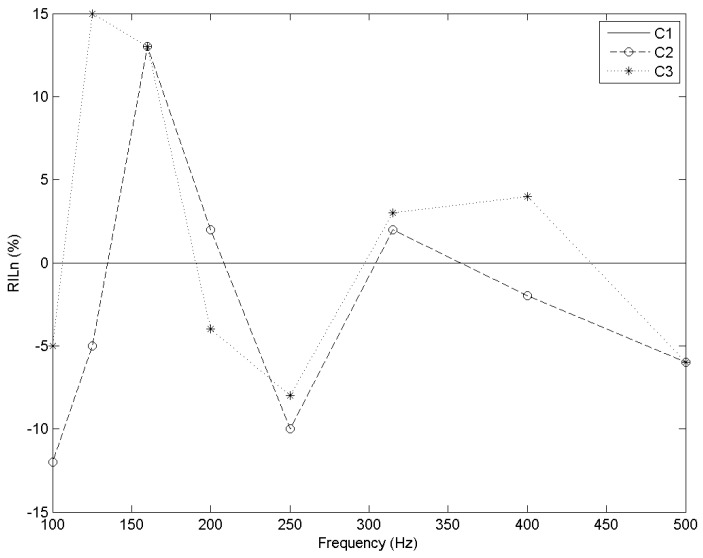
RIL_n_ (%) vs. frequency (Hz) for each cell type. Frequency range 100–500 Hz.

With regard to impact sound insulation, C2 and C3 have a similar response for frequencies approximately between 100 and 190 Hz, providing a better insulation than C1—up to 15% in the case of C3 (Figure 11).

**Figure 12:**
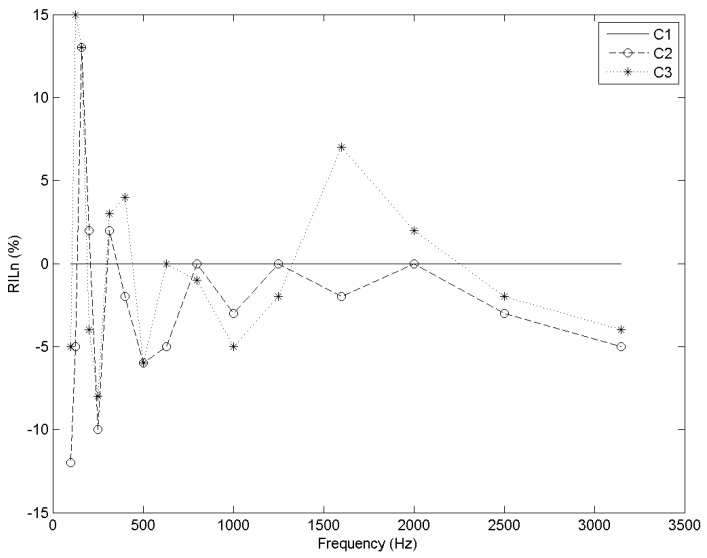
RIL_n_ (%) vs. frequency (Hz) for each cell type.

From approximately 190 to 290 Hz, both cells had a worse response than the standard—up to 10% (Figure 11). From 290 Hz and on, C2 is essentially less insulant than C1. However, from approximately 1300 to 2250 Hz, the performance of C3 was up to 7% better (Figure 12).

## 4. Conclusions

Based on the research presented here, some remarkable conclusions could be drawn.

Based on previous works, building elements could be doped with waste-tire rubber up to 20%. Elements with the maximum percentage of rubber in their composition (20%) provide a better response than the standard (0%), and the intermediate doped elements (10%) for low frequencies up to 140 dB in the case of airborne insulation, and 190 dB in the case of impact sound insulation. In the first case, the improvement was up to 50%, whereas in the second case, this percentage was hardly 15%. Therefore, highly doped construction elements are convenient to isolate very low frequency sounds, such as low frequency instruments (bass guitar, bass drum, etc.), low frequency road traffic vehicles (heavy trucks, tractors, etc.), barking dogs, greencuts, and chainsaws.

For middle and high frequencies, highly doped materials are generally less insulant than traditional materials. Intermediate doped materials show a more satisfactory result, although very close to the standard. Therefore, the intermediate doped and standard solutions are better to insulate average tone human voices, baby crying, vacuum cleaners, phones, airplanes, and low frequency motorbikes.

An explanation for this behaviour can be found in the interface generated between mortar and rubber. In a previous work [6], electronic microscopy showed a lack of interconnection in the interface created between mortar and rubber when concrete doped with waste-tire rubber is manufactured. The microcracks generated in this interface were able to soften and even absorb the effect of low frequencies, whereas high frequencies were only slightly affected. The increase of the percentage of rubber increments the presence of microcracks, so the displacement of sound pressure is allowed. Actually, material mixtures with a 10% and 20% of waste-tire rubber are around a 5% and a 10% less dense than conventional concrete, respectively. The reduction in density is due to the emergence of these microcracks, which encapsulate air. So, it could be stated that air–rubber and rubber–concrete borders of the microcracks encapsulate low frequency sounds, but are mainly effectless for high frequency sounds. Another point of interest is the ratio of density reduction versus waste-tire rubber. A correlation of 1 to 2 could be observed in this research. Nevertheless, this value is probably dependent on the manufacturing process of the building elements. In this work, vibro-compaction and slow curing were used. Both processes facilitated the expansion of the rubber inside the mixtures. Nevertheless, it might be profitable to investigate the influence of the manufacturing process in the acoustic response of the building elements.

Considering these conclusions, highly doped materials could be especially profitable in the building of auditoriums and highway sound barriers. Together with the insulation, the amount of recycled rubber in such bricks could be very positive for the environment.

On the other hand, intermediate doped materials could be used to build domestic constructions. Their insulant properties equal standard materials, and still offer a good opportunity to recycle waste-tire rubber.

## Figures and Tables

**Table 1:** Composition and density of mixtures.

Materials	M1	M2	M3
Waste-tire rubber (%)	0	10	20
Average dry density (kg/m^3^)	2036.8	1930.3	1847.5

**Table 2:** Composition of the building elements.

Cell Type	Mixture				
	Bricks	Hollow Blocks	Lattice Joists	Support Walls	Concrete Mortar
C1	M1	M1	M1	M1	M1
C2	M2	M2	M1 + rubber layer	M1	M1
C3	M3	M3	M1 + rubber layer	M1	M1
